# Competition and physician-induced demand in a healthcare market with regulated price: evidence from Ghana

**DOI:** 10.1007/s10754-021-09320-7

**Published:** 2021-12-17

**Authors:** Adolf Kwadzo Dzampe, Shingo Takahashi

**Affiliations:** Graduate School for International Development and Cooperation, Hiroshima University, 1-5-1 Kagamiyama, Higashi-Hiroshima, 739-8529 Japan

**Keywords:** Physician-induced demand, Doctor-to-population ratio, Regulated price, Competition, Hypertension, Ghana, I11

## Abstract

Using panel data of administrative claims spanning 36 months (2017–2019) and an instrumental variable method, this study examines whether physician-induced demand for hypertension disease care exists in Ghana’s healthcare system where price is regulated, and there is no co-payment. We find that an increase in competition—measured as a high doctor-to-population ratio at the district level—leads to an increase in the number of physician visits, suggesting physician-induced demand exists, and that effects are greater for large hospitals and public health providers. This result is further supported by alternative measures and specifications showing that physicians’ revenue from medication and gross revenue increase as the physician density increases. These pattern suggest that physicians in high density areas, faced with a decrease in number of patients per physician, make up for the decline in income by inducing more patient visits.

## Introduction

Doctors are often more informed about their patients’ health than the patients themselves. It is possible that, in order to increase their income, doctors advise their patients to consume more healthcare than the doctors themselves believe is necessary. This type of excess demand for healthcare is termed physician-induced demand (PID) (McGuire, 2000). The empirical debates over whether doctors are indeed inducing demand for their services has been a longstanding one.

A large corpus of empirical literature tests whether there exists PID (hereafter, PID hypothesis), but offers mixed results.^1^ The most common approach is to examine the effect of doctor-to-population ratio on the use of health service (Birch, 1988; Cromwell & Mitchell, 1986; Fuchs, 1978; Rossiter & Wilensky, 1983; Sekimoto & Ii, 2015; Sørensen & Grytten, 1999; Stano et al., 1985), where a positive relationship between the doctor-to-population ratio and the use of health service implies supplier-induced demand.

For example, Stano et al. (1985) use claims data of the Blue Cross-Blue Shield of Michigan and find a statistically significant and positive relationship between the physician density and per capita use of healthcare. Cromwell and Mitchell (1986) find a positive, albeit weak, relationship between use of elective surgery and supply of surgeons. Similarly, Fuchs (1978) find that per capita use of surgery increases by 3% when the surgeon-to-population ratio increases by 10%. Sekimoto and Ii (2015) use data on diabetes and hypertension patients to show a positive association between clinic physician density and frequency of physician visits, implying the existence of PID in Japan’s healthcare system.

Other studies find no PID in healthcare (Carlsen & Grytten, 1998; Sørensen & Grytten, 1999). For example, Carlsen and Grytten (1998) examine the relationship between population-to-physician ratio and number of visits to doctors as well as provision of laboratory services using a dataset of contract physicians in Norway, but find no evidence of inducement. Similarly, Sørensen and Grytten (1999) also find no evidence of PID in Norway, while Escarce (1992) find that higher supply of surgeons increases first-occurrence demand, but not the intensity of care demand in France. He concludes that improved access and stronger preferences may be a more likely explanation than PID.

Some studies also examine the effect of doctor density on the revenue or earnings of healthcare providers as a way of identifying PID. In the absence of inducement, and where fees are fixed, an increase in doctor density should decrease the revenue of doctors since their workload decreases. However, in the presence of inducement, a physician’s earning can increase. One possible mechanism is cross referral of patients among doctors, as Xirasagar and Lin (2006) note. As such, a number of studies have found a positive correlation between doctor density and doctor revenue (Tsai et al., 2004; Xirasagar & Lin, 2006).^2^

The literature shows that payment systems influence physician behaviour.^3^ For example, fee-for-service is associated with overutilization of medical services. This is because health providers are reimbursed for the quantity of services provided and not the appropriateness of care. On the other extreme end, capitation (where providers are paid a fixed, up-front rate per capita, regardless of the number of services used) provides incentive for under-provision of medical services (Eggleston, 2005; Ellis & McGuire, 1986; McGuire, 2000). In Ghana, consultations are reimbursed using the “Ghana Diagnosis Related Groups”^4^ method where fixed fee per outpatient visit is paid to the health provider, and medications are reimbursed by fee-for-service (Dzampe & Siita, 2020). Thus, Ghana’s health care payment system is likely to incentivize physician-induced demand.

The goal of this study is to investigate whether PID exists in Ghana’s healthcare system. Ghana provides an interesting econometric case to study PID. In 2003, Ghana introduced the National Health Insurance Scheme (NHIS), under which the price of health services is set by the government. Health providers consider prices to be low, and this low price has been a source of disagreement between the government and service providers for many years (Makinen et al., 2011). The regulatory environment was also eased to lower entry barriers for new health providers. For example, new health providers that fail to meet some of the regulatory requirements could receive provisional license (credentialing) and are able to start operations while working toward meeting them (Lamptey et al., 2017). In this environment, facing low prices and increased competition, doctors may face a strong incentive to induce patients to consume more healthcare services than necessary. Despite this possibility, there is almost no academic research that provides empirical evidence for whether PID indeed exists in Ghana. The only exception is Amporfu (2011), who finds no evidence of inducement in private hospitals in general, but does observe PID for the active population (18–60 years). This study, however, is limited to four hospitals in one district.

We use NHIS administrative claims data over a three-year period. For testing the PID hypothesis, we choose hypertension diseases. The treatment for hypertension provides doctors much scope for discretion, and medications are many and varied (Weber et al., 2014). Thus, the opportunity for PID is likely to be high. For example, when a patient presents a hypertension case, the doctor has several classes of antihypertension medications at their disposal to choose from.^5^ The doctor decides what combination of antihypertensive medicines to use and when to use it. It is this level of discretion that makes PID very likely (Cromwell & Mitchell, 1986). Given that hypertensive diseases are life-long diseases and are projected to increase among the population, especially in developing countries such as Ghana (Opoku et al., 2020), PID should be of major concern to policymakers.

The method we use follows the trend of past studies—we examine the relationship between the district-level doctor-to-population ratio and the service provider level use of hypertension care. A positive correlation is taken as supporting the existence of PID. We deal with the potential endogeneity of the doctor-to-population ratio using an instrumental variable method, where we use the size of the district population and the proportion of men in this population as instruments. Doctor-to-population ratio may be endogenous due to omitted factors such as average travel time or the general health knowledge of the population. Our instruments should affect the demand for health care only through doctor-to-population ratio. The two instruments, district population size and the male population ratio, are meant to capture the attractiveness of a location to doctors. In Ghana, districts with mining and commercial cocoa farming activities have vibrant local economies and high purchasing power (Chuhan-Pole et al., 2015) that makes them attractive locations for doctors. We use male population ratio as proxy for these locations because workers at mining or commercial cocoa farming areas in Ghana are predominantly male^6^ (Calys-Tagoe et al., 2015; Chuhan-Pole et al., 2015). At the same time, there is no reason to believe that male population ratio might directly affect the omitted travel time or the general health knowledge.

District population captures greater availability of amenities such as availability of good schools for doctors’ children, stable provision of electricity and water, and the existence of high-economic activities. Greater amenities may affect the doctor-to-population ratio, but may not have a direct effect on the demand for healthcare (Cromwell & Mitchell, 1986; Fuchs, 1978; Jiang & Begun, 2002; Peacock & Richardson, 2007). According to Peacock and Richardson (2007), an unpleasant location represents high cost which will reduce doctor supply. Carlsen and Grytten (1998), Peacock and Richardson (2007), and Sørensen and Grytten (1999) also used district population and variables that capture high economic activities as instruments for doctor-to-population ratio.

One advantage of the Ghana data is that they allow us to counter some common problems faced in doctor-to-population ratio studies. First, patients in areas with less physician density may travel to areas with high physician density for treatment, especially where increased densities result in higher quality competition (Dranove & Satterthwaite, 1992), causing a difficulty in identifying the existence of PID. To avoid this problem, Dranove and Wehner (1994), in their study of PID for childbirths in the US, define larger geographical areas for analysis. In Ghana, such a border crossing concern is negligible owing to its poor transportation system.

Second, increased doctor density increases the use of health care services owing to the reduced denial of care (rationing) due to the greater availability of medical services. As such, a positive relationship between doctor density and use of health care services may reflect the reduced rationing rather than doctor-initiated demand (Carlsen & Grytten, 1998; Escarce, 1992; Reinhardt, 1985; Sørensen & Grytten, 1999; Stano, 1985). However, in Ghana, rationing by health providers is difficult owing to its significant monitoring and control system. For example, the NHIA has contractual agreements with health providers prohibiting rationing, among other things (Wang et al., 2017). There are established customer service call centers for NHIS patients to report any dissatisfaction with or denial of healthcare. Birch (1988), for example, also note similar contractual constraints on dentists in the UK which prevented them from engaging in rationing, and further point out that such a practice would be contrary to professional ethics.

Third, in an environment where doctors can set the price for a treatment, increased competition leads to reductions in the price, which, in turn, increases service use, making it difficult to distinguish this price effect from the PID effect. However, Ghana’s NHIS sets the price for each treatment, thus making the identification of PID straightforward, since the price effect is absent.

The remaining paper is organized as follows: Sect. 2 describes the context of the study and presents a brief background about the healthcare system and health insurance in Ghana. Section 3 describes the theoretical model that motivates our empirical strategy. The empirical implementation strategy, including data, variable description, and descriptive statistics are presented in Sect. 4. Section 5 presents the results, and in Sect. 6 we have discussion and conclusion.

## Background

Ghana introduced the NHIS in 2003 to remove financial barriers to access to healthcare. NHIS patients do not pay any fees at the point of service and can access healthcare for over 95% of disease conditions. Children under 18 years, adults 70 years or older, pregnant women, and all active social security contributors are all exempted from payment of premiums. Fees are pre-determined by the regulator, National Health Insurance Authority (NHIA). As of 2019, the scheme had about 12.2 million (NHIA, 2020)—about 40% of the population.

Doctors are free to set up health facilities anywhere and need to sign a contract with the NHIA to provide services to NHIS members. Also, cross referrals among doctors are possible, and within-hospital referral and hospital-based outpatient services (without referrals) are permitted under the NHIS (Wang et al., 2017). Primary care is thus accessible at all levels (including clinics to large hospitals) of the healthcare system in Ghana. At the same time, regulatory requirements^7^ for setting up a health facility in Ghana have been eased to encourage more private practice. For example, health providers can receive provisional license (credentialing) when they fail to meet the regulatory requirements. This enables them to start operations while working toward meeting them (Lamptey et al., 2017). It is common for patients to be asked to return for multiple reviews within short periods of time, yet with little evidence of what was done on each visit when patient records are audited (NHIA, 2013). One possible reason is that medical literacy rates are quite low (Amoah, 2018), and so one may expect patients to be generally passive. Note that this makes it easier for doctors to induce demand.

Some service providers (public and private) rely heavily on NHIS for revenue—as high as 80% in some cases (NHIA, 2013; Wang et al., 2017). These revenues constitute a significant part of allowances and other “compensations” for doctors and other staff.^8^ Unfortunately, reimbursements of claims have been erratic and characterized by long delays over the past ten or more years. As of 2009, the NHIS was in a deficit (reaching GHc 300 million by 2014)^9^ (Wang et al., 2017). According to Wang et al (2017), between 2005 and 2014, claims expenditures had risen from just about GHc 7.6 million to over GHc1.1 billion, respectively. The escalating cost of healthcare has been a major concern for the government. The belief was that introducing competition among doctors would bring about greater efficiency. However, it seems plausible that, in an environment characterized by long delays in reimbursement (Wang et al., 2017), low price (Makinen et al., 2011), and poorly informed patients (Amoah, 2018), greater competition may be a logical incentive for inducement.

## Model

To provide a foundation to our empirical strategy, we review the most basic model of PID described by McGuire (2000). According to McGuire (2000), PID is defined as excess demand that results when a physician influences a patient’s demand for care against the physician’s own interpretation of the best interest of the patient. Thus, not all influence comprises inducement, because, by definition, physicians use their expertise to influence patients’ actions to improve their health. Thus, PID must be defined as “undue influence.” Physicians have a number of ways to unduly influence patients to increase the use of care. For example, they might find that the health condition of the patient is favorable, but may hide this information. The physician may also persuade the patient to believe that their state of health is worse than it actually is, and thus advise the patient to consume more medical care or medication.

In McGuire’s (2000) study, the physician’s utility function has two augments: income and inducement. The “undue influence” characteristics of inducement are captured by the negative association between inducement and the physician’s utility, assuming there is a psychic cost to unduly influencing patients. Now, consider that the physician increases the inducement by one unit. This will increase their income through an increase in the medical care that the patient consumes, which, in turn, increases their utility. On the other hand, the increase in inducement directly reduces their utility owing to its psychic cost. The optimal inducement is thus decided where the marginal increase in utility that arises from an increase in income is just equal to the marginal decrease in utility that results from the psychic cost of inducement.

More formally, McGuire’s (2000) model is written as follows:

1 $${\text{Max}}\; U = U \left( {Y,I} \right)$$

2 $${\text{Where}}\;Y = N \left( {m_{1} x_{1} \left( {i_{1} } \right) + m_{2} x_{2} \left( {i_{2} } \right)} \right)$$

3 $$I = N \left( {i_{1} + i_{2} } \right)$$

where $U$ is the physician’s utility, $Y$ is income, and $I$ is the total inducement. For generality, this model allows physician to provide two types of care, ${x}_{1}$ and ${x}_{2}$ though a model with single type of care model can capture the nature of inducement. For each type, the physician determines the level of inducement ${i}_{1}$ and ${i}_{2}$. The price of each type is ${m}_{1}$ and ${m}_{2}$, respectively, and $N$ is the number of patients that the doctor sees, which is exogenously given. This model assumes ${U}_{Y}>0;$ ${U}_{YY}<0;$ ${U}_{I}<0;$ ${U}_{II}<0.$ The first-order condition is then given by

4 $$m_{1} x^{\prime}_{1} \left( {i_{1} } \right) = m_{2} x^{\prime}_{2} \left( {i_{2} } \right) = \frac{{ - U_{I} \left( {Y,I} \right)}}{{U_{Y} \left( {Y,I} \right)}}$$

Thus, the number of patients, $N$, affects only the third term, $\frac{{-U}_{I}(Y,I)}{{U}_{Y}(Y,I)}$. Suppose there is an exogenous decrease in the number of patients. How does this change affect the demand for physician services, ${x}_{1}\left({i}_{1}\right)$ and ${x}_{2}\left({i}_{2}\right)$? The reduction in $N$ reduces $Y$ through constraint *(2)*, which increases ${U}_{Y}(Y,I)$. It also reduces $I$ through constraint *(3)*, which reduces ${-U}_{I}(Y,I)$. Thus, the third term, $\frac{{-U}_{I}(Y,I)}{{U}_{Y}(Y,I)}$, decreases. To regain equality, $Y$ must increase, along with *I*. This increase in $I$ will then increase the demand for physician services, ${x}_{1}\left({i}_{1}\right)$ and ${x}_{2}\left({i}_{2}\right)$.

Intuitively speaking, when the number of patients decreases, it reduces the physician’s income, which reduces their utility. At the same time, it reduces the total psychic costs of inducement because the total inducement reduces, which provides more room for the physician to engage in inducement per patient. The physician will, thus, increase inducement per patient as long as the marginal decrease in utility from the additional inducement is smaller than the marginal gain in utility from the additional income. The increase in the inducement per patient will then increase the demand for the physician. Most importantly, the testable prediction of this theory is that, if inducement exists, an exogenous decrease in the number of patients will increase the demand for medical care.

## Methodology and data

### Empirical method

As detailed in the previous section, if there exist PID, an exogenous decrease in the number of patients will increase the demand for medical care. To test whether this prediction is supported by the data, we examine if the district-level physician-to-population ratio positively correlates with the service provider level use. We focus on the use of service for hypertension, since its treatment provides doctors with greater discretion and, thus, more opportunity for PID.

Consider the following model:

5 $$log(Visits_{it} ) = \beta_{0} + \beta_{1} log(Doctor/Population_{ct} ) + X_{it} \Pi + Z_{ct} \Gamma + \lambda_{t} + \alpha_{i} + \varepsilon_{it}$$

The dependent variable ${Visits}_{it}$ is the number of visits to provider *i* in time *t*, where *t* ranges over 36 months from 2017 to 2019. ${Doctor/Population}_{ct}$ is the district level doctor-to-population ratio, the main variable of interest. If PID exists, the coefficient on this variable, ${\beta }_{1}$, will be positive. ${X}_{it}$ is a vector of provider-level controls, such as the median age of patients, type of health provider, ownership status, and prescribing level of the health provider. ${Z}_{ct}$ is a vector of district-level controls, namely, the proportion of population aged 65 years and above and the availability of complementary staff such as nurses and alternative source of care such as midwives in a district. We include provider fixed effects ${\alpha }_{i}$, to capture time invariant provider level characteristics that may be correlated with the doctor to population ratio. For example, hospitals with a higher reputation may be more concentrated in high doctor density area. At the same time, hospital reputation would attract more patients. Inclusion of provider level fixed effects will remove the bias that stems from this type of endogeneity. ${\lambda }_{t}$ captures month effects. Furthermore, we estimate Eq. (5) by replacing the dependent variable with the logarithm of revenue for medication and of gross revenue as a robustness check.

Even after controlling for provider level fixed effects, there may still be endogeneity concern for the use of district-level physician-to-population ratio due to time varying unobserved variables, as studies already note (Carlsen & Grytten, 1998; Cromwell & Mitchell, 1986; Escarce, 1992; Peacock & Richardson, 2007; Sørensen & Grytten, 1999). For example, in a district where the doctor-to-population ratio is high, the average distance that a patient needs to travel to see a doctor may reduce. This reduced travel time is likely to positively affect the demand for physician services.^10^ Since travel time negatively correlates with the demand for care and with the doctor-to-population ratio, there will be a positive bias in the coefficient for the doctor-to-population ratio.

Second, the general health knowledge of patients is also of concern. For example, in metropolitan areas or places with high doctor density, people may generally have more access to health information and, thus, may have better knowledge of maintaining health through lifestyle choices (Lönnberg et al., 2020; Wu et al., 2017). Such people are less likely to seek medical care. This behavior is expected to introduce a negative bias in the doctor-to-population ratio coefficients.

To deal with the endogeneity that stems from time varying unobserved variables, we use the instrumental variable method. Our instruments should affect the demand for healthcare only through the doctor-to-population ratio. Thus, we use variables that capture the “desirability of location” for doctors, that is, district population size and male population ratio that capture greater availability of amenities such as availability of good schools for doctors’ children, stable provision of electricity and water, and the existence of high-economic activities, as discussed in previous session.

### Data, variables, and summary statistics

The data come from several sources. The main data are obtained from the claims database of the NHIA.^11^ These claims contain information on patients and cost of treatment. Specifically, they contain information on age, date of treatment, diagnosis, the International Classification of Diseases (ICD-10) codes, type of treatment, and information on charges for services and medicines. Data on the characteristics of service providers are also obtained from the NHIA, which include the type of health provider, ownership, prescribing level, and location of health facility, making it possible to link the district-level data with the service provider-level data.

We obtain the claims data for the period 2017–2019, which yields 20 million observations. This is period when reliable data became available due to the introduction of electronic databases. Before 2017, data were fragmented and unreliable. It also represents a good milestone (15 years) since the introduction of NHIS – a long enough period for health providers and the public to be well familiar with the NHIS. Our focus on hypertension, however, reduces the number of observations considerably. We identify hypertensive disease cases by the ICD-10 codes^12^ of I10, I11, I12, I13, and I15, and, in few cases, based on the descriptions used instead of ICD-10 codes.^13^ In all, about 2 million hypertensive disease cases are identified, of which about 1,762,015 are outpatient cases. We focus on patients who were 18 years old or above at the time because pediatric hypertension is much more difficult to diagnose accurately, and its prevalence rate is low (Hansen et al., 2007). After cleaning the data, we retain about 90% of the observations for our analysis, which are used to construct panel data for 243 providers from 160 districts over 36 months. Data for the independent and location variables are obtained from two sources: The district population and sub-populations estimates are obtained from the US Census Bureau,^14^ while the number of doctors, midwives, and nurses in each district were purposely compiled from the personnel database for this study by the Monitoring and Evaluation Unit of the Ministry of Health.

Table 1 shows the variables with their summary statistics. The main dependent variable is the number of patient visits per month to each health provider. As a robustness check, we use two alternative dependent variables, *namely*, the revenue from hypertensive medications, and the gross revenue from treatment including consultation fees. All the dependent variables are at the provider level, and all monetary values were adjusted for inflation with 2018 as the base year.

**Table 1:** Summary statistics

Variable	Mean	Standard deviation
Number of visits per month	519.889	520.936
Revenue from hypertensive medication	15,668.92	24,373.715
Gross revenue from hypertensive care	23,476.389	33,417.709
Median age of adult patients in a month	62.424	3.24
Health facility is government owned	.311	.463
Health facility is not-for-profit	.416	.493
Health facility is for-profit	.273	.446
Health provider is a health centre	.189	.392
Health provider is a clinic	.106	.308
Health provider is a primary hospital	.645	.479
Health provider is a secondary hospital	.033	.179
Health provider is a tertiary hospital	.027	.161
Medicine prescribing level M1	.007	.081
Medicine prescribing level B1	.291	.454
Medicine prescribing level B2	.155	.362
Medicine prescribing level C1	.529	.499
Medicine prescribing level D1	.019	.135
Doctors per 10,000 residents	2.189	2.595
Proportion of population aged 65 and over	.043	.012
Registered nurses per 10,000 residents	11.557	9.46
Community health nurses per 10,000 residents	6.273	3.822
Midwives per 10,000 residents	5.447	3.451
Total district population	429,187.6	634,804.54
Proportion of men in the district	.49	.013
N	3382	

The revenue from hypertensive medication captures intensity of care. For hypertension care, a patient is usually given a fixed unit of medication per day. Thus, the number of units of medication a patient is give per month does not necessarily vary with the number of visits that the patient makes. As such, other things being equal, an increase in the revenue from medication for hypertension care is likely to indicate that the daily dosage of medication per patient increased or another class of hypertension medicine was added. Thus, this variable captures PID through the possible manipulation of intensity of care.^15^ The gross revenue provides an alternative way to capture the overall increase in the use of health care stemming from an increase in visits, an increase in the intensity of care, or an increase in the number of patients. As Xirasagar and Lin (2006) showed, doctors can manipulate the number of patients through cross referral of patients.

The mean of visits per month per provider is about 520. The mean revenue from medications is about GHc 15,668, and the mean total gross revenue is about GHc 23,476 per month. Our focal explanatory variable is the doctor-to-population ratio, measured as the number of doctors per 10,000 residents in a district. Overall, the mean ratio is about 2. The data also show that areas with doctor density ratios greater than 6 are mostly metropolitan areas, such as Accra and Tamale.^16^ This reflects the uneven distribution of doctors in favor of metropolitan areas. In addition to the median adult age, we include the proportion of people aged 65 years and above as a measure of the health status or “predisposing health indicators,” following Dranove and Wehner, (1994).

To control for variations in inputs such as nurses (who provide supporting and complementary works to doctors), we include the number of registered nurses per 10,000 residents in the district. In most rural areas, midwives and community health nurses also serve as alternative sources of care for patients and thus, we additionally control for the number of community health nurses and the number of midwives per 10,000 residents in the district. Finally, to control for the characteristics of the health provider, we include ownership, type of health provider, and the level of prescribing medicine by the health provider.

**Fig. 1:**
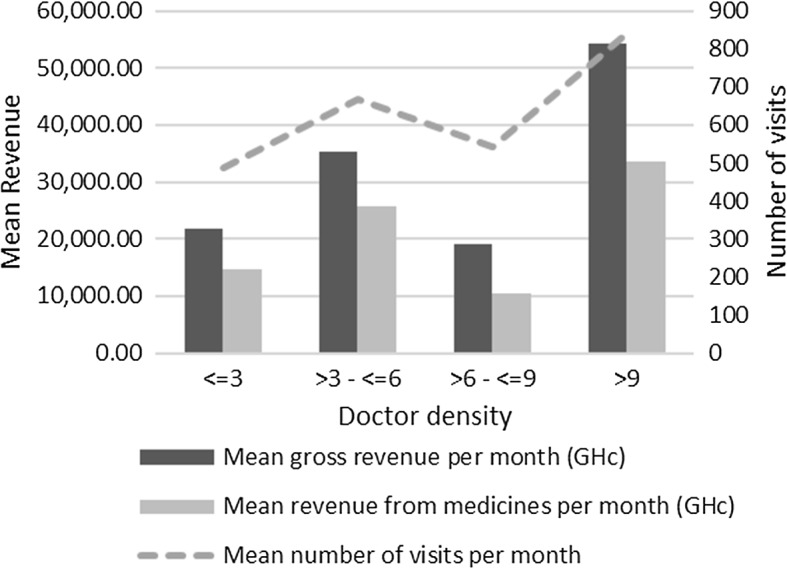
Relationship between doctor density and mean number of visits, mean revenue from medicines and mean gross revenue per month

Figure 1 shows that the average number of visits and revenues increase with the doctor to population density. For example, the average visits per provider is higher for high-density areas (840 visits) than low-density areas (488 visits). Similarly, revenue from medications per month is higher for high-density areas (GHc 33,696) than for low-density areas (GHc 14,716). The mean overall revenue in high-doctor density areas is about GHc 54,153 compared with GHc 21,757 in low-doctor density areas.

## Empirical results

### Main results

Column (1) of Table 2 presents the results of the effect of the doctor-to-population ratio on the number of visits per month per provider using pooled ordinary least squares (OLS). The results imply a positive associate between the doctor-to-population ratio and the number of visits, which support the hypothesis that PID exists. A 1% increase in the doctor-to-population ratio is associated with a 0.35% increase in the number of visits, which is evidence of PID. However, as noted earlier, the doctor-to-population ratio may still be endogenous even after controlling for the provider level fixed effects.

**Table 2:** OLS and FE-2SLS regressions for total visits, revenue from medications, and gross revenue

	(1)	(2)	(3)	(4)	(5)	(6)
Dependent variable	log(number of visits per month)		log(revenue from hypertensive medications)		log(gross revenue from hypertensive care)	
	OLS	FE-2SLS	OLS	FE-2SLS	OLS	FE- 2SLS
Doctors per 10,000	.345***	.751***	.4***	.578*	.386***	.487*
Residents	(.033)	(.277)	(.051)	(.323)	(.04)	(.272)
Registered nurses per	− .364***	− .007	− .563***	.313***	− .536***	.102
10,000 residents	(.062)	(.097)	(.099)	(.116)	(.075)	(.097)
Community health nurses	.139***	− .113	.162**	.37**	.159**	.011
Per 10,000 residents	(.053)	(.133)	(.074)	(.146)	(.064)	(.128)
Midwives per 10,000	− .346***	− 1.747***	− .129	− 1.615***	− .171**	− 1.428***
Residents	(.068)	(.464)	(.101)	(.57)	(.081)	(.464)
Proportion of population	.626***	− 17.51	.679***	60.654**	.715***	34.124
Aged 65 and above	(.092)	(23.44)	(.117)	(28.434)	(.106)	(24.325)
Median age	− .033***	.001	− .023**	.004	− .028***	− .001
	(.007)	(.006)	(.01)	(.008)	(.008)	(.006)
*Health provider type (base: primary hospital)*						
Health center	− .317*	−	− 1.312***	−	− .862***	−
	(.185)		(.213)		(.202)	
Clinic	− .128	−	− .427***	−	− .273***	−
	(.111)		(.098)		(.1)	
Secondary hospital	− .122**	−	− 2.201***	−	− .799***	−
	(.062)		(.169)		(.084)	
*Ownership of health facility (base: government)*						
Not − for − profit provider	− .181***	−	− .505***	−	− .393***	−
	(.055)		(.086)		(.068)	
For − profit provider	− 1.039***	−	− 1.811***	−	− 1.141***	–
	(.085)		(.117)		(.102)	
*Medicine prescribing level of health facility (base: level C)*						
Prescribing level M1	− 1.152***	−	− .385	−	− .751***	−
	(.248)		(.295)		(.278)	
Prescribing level B1	− 1.31***	−	− 1.533***	−	− 1.61***	−
	(.18)		(.213)		(.197)	
Prescribing level B2	− .862***	−	− .291*	−	− .758***	−
	(.164)		(.176)		(.173)	
R − squared	.59	−	.612	−	.646	–
Month dummies	YES	YES	YES	YES	YES	YES
Overid (J statistic)	–	0.604	–	0.018	–	1.459
Underid (χ2 (2))	−	69.4	−	68.8	–	69.4
Weakiv (*F* statistic)	−	31.32	−	31.13	–	31.32

Robust standard errors are in parentheses. Revenue values are adjusted for inflation (base year 2018). The following variables are time-invariant: type of health provider, ownership of health facility, and prescribing level of health facility. Independent variables are log transformed except median adult age, categorical and dummies variables

^***^p < .01, **p < .05, *p < *.1*

Thus, in Column (2), we apply the two stage least square estimation where the instruments are district population size and proportion of men in the district. This is our preferred model. Note that, in addition to applying instrumental variables, this estimation also removes the provider level fixed effects. Specifically, we demeaned all the variables, and then applied demeaned instrumental variables. First, note that we failed to reject the overidentifying assumptions under the null hypothesis that the instruments are exogenous (see Hansen’s *J* statistics at the bottom of Table 2). Second, we perform the under-identification test of the relevance of instruments, and rejected the null hypothesis. We also perform the test of weak identification. A widely accepted rule-of-thumb criterion for a strong instrument is an *F*-statistic greater than 10 reported in the first-stage regression (Staiger & Stock, 1997). The *F*-statistic is above 31, indicating that our instruments are strong. Both instruments are highly statistically significant in the first-stage results with the expected positive signs. All these diagnostic tests further increased our confidence in the validity of the choice of our instruments.

From the FE-2SLS estimates in column (2), we find that the doctor-to-population ratio has a significant positive effect on the number of visits, which support the hypothesis that PID exists. A 1% increase in the doctor density leads to an increase in the number of visits by 0.75%, an elasticity estimate consistent with the PID literature (Peacock & Richardson, 2007). Our finding of the evidence of PID is not necessarily surprising, since health providers consider the prices set by NHIA to be too low and have openly disagreed with the government over this issue for many years. Since prices are fixed, health providers can only influence quantity. Thus, when the number of physicians increase, to make up for the lower number of patients and maintain their income, they induce more visits. The relatively large elasticity for the doctor-to-population ratio may be reflecting the perceived low price of healthcare providers coupled with long delays in the reimbursement of claims. In such an environment, competitive pressures may lead health providers to induce many more visits to maintain their incomes. The fact that Ghana is less developed country and patients are less informed and passive would have made the inducement all the more likely.

### Robustness checks and sensitivity analysis

To find further support for our inducement hypothesis, we estimate models with two alternative dependent variables, the revenue from hypertensive medication, and the gross revenue. We also perform sub-sample analyses to evaluate the sensitivity of our results. As noted, revenue for medication captures the intensity of care, while the gross revenue is an alternative way to capture the increase in the use of health care. In the absence of inducement, when the doctor-to-population ratio increases and the fees are fixed, we expect doctors’ earnings to fall as their workload reduces. However, if physicians engage in inducement by increasing the number of visits, increasing the intensity of care, or increasing the number of patients through cross-referral, doctors’ earnings can increase (Carlsen & Grytten, 1998; Sørensen & Grytten, 1999; Xirasagar & Lin, 2006).

Columns (3) and (4) in Table 2 use the revenue from medication as the dependent variable. Both models show a strong positive relation between doctor density and revenue from medications. For the pooled OLS estimation, a 1% increase in doctor density is associated with a 0.40% increase in revenue from medications. The corresponding elasticity for the fixed effect 2SLS is 0.58%. Thus, doctors are able to recover some lost revenue when physician-to-population ratio increases by increasing the intensity of treatment (i.e., prescribing more medications) or via increasing the visits. This increase in intensity of care cannot be attributed to the availability effect or a reduction in access cost, because increasing the number of medications is mainly the decision of physicians and thus reflects physician behavior. When rationing exists, revenue from medications is unlikely to increase with increased doctor density. The more likely explanation would be PID. We note that, for the 2SLS estimation, we failed to reject the overidentifying assumption and weak instruments assumption.

Columns (5) and (6) use the service provider level gross revenue as the dependent variable. Both models show a strong positive relationship between doctor to population density and the gross revenue, lending further support for the PID hypothesis. This result is consistent with the findings by Xirasagar and Lin (2006) for Taiwan. The elasticities for the pooled OLS estimation and 2SLS estimation with fixed effects are 0.39 and 0.49, respectively. Gross revenue can also be a measure of service use since it reflects the use of both services and fees. One problem that plagued the past studies was that, in some countries, fees can vary widely across health providers and thus gross revenue is a poor proxy for the use of health service. In Ghana, however, fees are regulated and do not vary across health providers. Thus, the increasing gross revenue is a good proxy for the use of health care services.

Table 3 presents the results of our sub-sample analyses where we first restrict our sample to large health providers (that is, providers categorized as district hospital or above with inpatient services). We expect that these hospitals would have greater capacity to induce demand, since they provide wider range of services (e.g., antihypertensive medications). From column (1) of Table 3 we find that the effect of competition on number of visits is much stronger among large hospitals (0.94%) than across all types of health providers.

**Table 3:** FE-2SLS regressions for sub-sample analyses of the effect of doctor density on number of visits

	(1)	(2)	(3)
	Large health providers	Public health providers	Excluding Accra Metro
	FE-2SLS	FE-2SLS	FE-2SLS
Doctors per 10,000	.944***	1.426***	.783**
Residents	(.27)	(.302)	(.345)
Registered nurses per	.008	.095	− .018
10,000 residents	(.132)	(.093)	(.104)
Community health nurses	− .065	.965***	− .094
Per 10,000 residents	(.152)	(.299)	(.14)
Midwives per 10,000	− 1.942***	− 2.292***	− 1.812***
Residents	(.435)	(.496)	(.587)
Proportion of population	− 14.5	83.118***	− 19.588
Aged 65 and above	(27.279)	(25.281)	(27.235)
Median age	.012	.015	.001
	(.011)	(.012)	(.007)
Month dummies	YES	YES	YES
Overid (J statistic)	2.022	0.114	0.536
Underid (χ2 (2))	52.82	85.75	47.05
Weakiv (*F* statistic)	23.93	32.70	22.11
N	2293	992	2852

Robust standard errors are in parentheses. The following time-invariant variables were dropped: type of health provider, ownership of health facility, and prescribing level of health facility. All variables are log transformed except median adult age, categorical and dummies variables

^***^ p < .01, ** p < .05, * p < *.1*

Next, we restrict the sample to public providers. Contrary to the believe that public health providers may have little financial incentive to induce demand, there are reasons why they may have equal or even greater incentive to induce demand in Ghana. Firstly, public providers are increasingly reliant on the NHIS for revenue for their operations (Wang et al., 2017), including paying allowances of doctors (McCoy et al., 2008), as noted earlier. Secondly, unlike private providers that could recover some lost revenue through other means like charging patients out-of-pocket, public providers face greater public backlash for doing so. Thus, their revenue is likely to be more reliant on patients visits, which in turn provides strong incentive to induce demand. Column (2) indeed show a much stronger effect (1.43%) than across all types of ownership.

Finally, we exclude Accra metropolitan area, located in the national capital (where the bulk of doctors are located, and patients are expected to be better informed) to examine inducement behavior where information asymmetry may be greater. From column (3) of Table 3, we find that the effect was slightly higher (0.78%).

## Discussion and conclusion

The presence of physician induced demand (PID) has been suspected in Ghana for long, where fees are fixed at extremely low price, patients face no cost at the point of service, doctors are free to set up service anywhere under low requirements, and the population are not sophisticated in terms of health care knowledge. This is obviously a fertile ground for physicians to induce demand. However, this is the first rigorous empirical study that investigates whether PID exists in Ghana. After accounting for the endogeneity of doctor-to-population ratio by using population size and proportion of district population male as instruments, we find evidence that physician induced demand is present in hypertensive disease care in Ghana. Our finding is consistent with many empirical studies that test the PID hypothesis (Birch, 1988; Delattre & Dormont, 2003; Peacock & Richardson, 2007; Stano et al., 1985; Xirasagar & Lin, 2006). In addition, we hypothesized that inducement is stronger for larger health providers that have greater capacity to induce demand, and for public health providers who are more reliant on the revenues from NHIS. We find supporting evidence for these additional hypotheses. Overall, our results strongly support the hypothesis that physician induced demand exists in Ghana.

Our findings have important policy implications. Evidently, increasing competition in an environment where prices are low and regulated and patients bear no cost at the point of service is likely to lead to inducement. Any policy directed at redistribution of doctors may thus be justified. However, simply easing regulatory requirements is unlikely to achieve the desired results, because doctors in high-density areas may be unwilling to move to low-density areas, where they cannot induce demand. One solution is to incentivize health providers to locate themselves in areas with low doctor densities. Provisional licenses may *only* be granted to such health providers. For example, in England physician contracts includes financial and other incentives for physicians locating in deprived areas (Gravelle et al., 2008). Health provider payment system reforms (such as capitation) as a way to influence physician behavior has been one common approach (Labelle et al., 1994). Alternatively, fees may be revised to reflect performance—a practical approach could be routine audits of random sample of claims under quality assurance program as proposed by Labelle et al. (1994). On the demand side, any regulated price needs to reflect the cost of care, and patients may need to incur some out-of-pocket expenses at the point of service.

An interesting area for future research is examining the implications of PID with respect to the quality of healthcare. Studies should examine the extent to which PID contributes to escalating healthcare expenditure in Ghana as well. Another question is whether there is induced demand for all disease conditions in Ghana, since inducement for hypertension diseases may not necessarily mean inducement of demand for all other disease conditions. As such, we are unable to generalize our results to other diseases conditions. However, this is a logical place to start, given that hypertensive diseases constitute a significant proportion of disease conditions among outpatient visits in Ghana and are projected to increase (Opoku et al., 2020).

## Data Availability

The data that support the findings of this study are available from third parties. Restrictions apply to the availability of these data, which were used under license for this study. Please contact the corresponding author for more information about data accessibility.

## Footnotes

1 For a survey of the literature, see Léonard et al. (2009).

2 Beside Bromberg and Price (1990) who review the role of supplier-induced demand in the rising cost of health care in South Africa, and Makinde (2016) who examine how financial incentives to physician by medical tourism centers may be the reason why medical tourism outside Nigeria is on the rise, studies that explicitly examine how competition influences the volume and intensity of medical services from an African context are virtually non-existent.

3 See McClellan (2011) and McGuire (2000) for reviews on payment systems and health provider behaviour.

4 The Ghana Diagnosis Related Groups method defines hospital products based on classes of patients receiving similar sets of services or resource use (Fetter & Freeman, 1986). Ghana extended this classification system to outpatient services, with reimbursements at a fixed fee per encounter.

5 Each class of medication has a different mechanism for controlling blood pressure and, therefore, could be used in several combinations. For example, diuretics help rid excess sodium, alpha blockers reduce artery resistance and relax muscles of the vascular wall, while calcium channel blockers prevent calcium ions from entering the smooth muscle cells of the heart and arteries, thereby reducing blood pressure. See Weber et al., (2014) for comprehensive guidelines on the various classes of antihypertensive medications and the many combinations in which they can be used in the management of hypertension.

6 Indeed, in our data, mining districts such as Tarkwa and Juabeso have higher male population ratios (52%) than the national average (49%), thus a good proxy for these areas with high economic activities not captured by district population. See https://www.statsghana.gov.gh for national statistics.

7 Health Institutions and Facilities Act, 2011 (ACT 829) and National Health Insurance Act, 2012 (ACT 852).

8 Only 26% of doctors’ monthly income comprises basic salary, with allowances constituting more than 60% (McCoy et al., 2008; Witter et al., 2007).

9 GHc refers to Ghana cedis.

10 More doctors may reduce the time price of care by reducing waiting time and travel time to see a doctor (Escarce, 1992), which are the two important factors that affect demand for health services (Phelps & Newhouse, 1974). Alternatively, patients in areas with high doctor density may develop a preference for seeking second opinions, while doctors in these areas may offer more amenities or high-quality care that patients value.

11 We merged the two electronic databases (*namely* E-claims and Claim-it) which the NHIA has been running since 2017 for its administrative purposes.

12 The ICD was developed to track diseases in the population and has been led by the World Health Organization since 1948 (Hirsch et al., 2016). Ghana uses the 2016 version of the ICD-10 codes; codes I10–I15 identify hypertension diseases as the primary diagnosis.

13 We select cases that sufficiently describe hypertensive diseases, such as “hypertension, essential hypertension, chronic hypertension, hypertension old, secondary hypertension and primary (essential) hypertension.” These diseases constitute only about 4% of the total data.

14 The US Census Bureau provides subnational population estimates for 2000–2020 in a geographic information system for users of the Spatial Data Repository, funded by the U.S. President’s Emergency Plan for AIDS Relief in collaboration with the Ghana Statistical Service.

15 For the evidence of PID through the choice of content or increase in number of prescription drugs per visit, see Delattre and Dormont, 2003 and Hu, Decker and Chou, 2017.

16 Consistent with the general distribution of doctors in Ghana, about 40% of doctors are in the Greater Accra Region, with 1 doctor for 3,052 residents, compared with the Upper East Region, where the doctor-to-population ratio is 1 to 26,489 (Ghana Health Service, 2018).

## Code availability

Code for data cleaning and analysis is available upon request.
